# Contribution of *Acinetobacter*-derived cephalosporinase-30 to sulbactam resistance in *Acinetobacter baumannii*

**DOI:** 10.3389/fmicb.2015.00231

**Published:** 2015-03-25

**Authors:** Shu-Chen Kuo, Yi-Tzu Lee, Tsai-Ling Yang Lauderdale, Wei-Cheng Huang, Ming-Fen Chuang, Chien-Pei Chen, Shey-Chiang Su, Kuan-Rong Lee, Te-Li Chen

**Affiliations:** ^1^Institute of Clinical Medicine, Schsool of Medicine, National Yang-Ming UniversityTaipei, Taiwan; ^2^National Institute of Infectious Diseases and Vaccinology, National Health Research InstitutesTaipei, Taiwan; ^3^Division of Infectious Diseases, Taipei Veterans General HospitalTaipei, Taiwan; ^4^Emergency Department, Taipei Veterans General HospitalTaipei, Taiwan; ^5^Department of Internal Medicine, Mackay Memorial HospitalHsin-Chu, Taiwan; ^6^Department of Molecular Medicine and Institute of Life Science, National Tsing Hua UniversityHsin-Chu, Taiwan

**Keywords:** sulbactam, mechanisms of resistance, *Acinetobacter baumannii*, transformation, *Acinetobacter*-derived cephalosporinase (ADC)

## Abstract

The sulbactam resistance rate in *Acinetobacter baumannii* has increased worldwide. Previous reports have shown that the β-lactamase *bla*_TEM-1_ confers resistance to sulbactam in *A. baumannii*. The purpose of this study was to examine whether other β-lactamases, including the *Acinetobacter*-derived cephalosporinase (ADC), OXA-23, OXA-24/72, and OXA-58 families, also contribute to sulbactam resistance in *A. baumannii*. The correlation between these β-lactamases and the sulbactam minimal inhibitory concentration (MIC) was determined using *A. baumannii* clinical isolates from diverse clonality, which were collected in a nationwide surveillance program from 2002 to 2010 in Taiwan. A possible association between the genetic structure of IS*Aba1*-*bla*_ADC-30_ and sulbactam resistance was observed because this genetic structure was detected in 97% of sulbactam-resistant strains compared with 10% of sulbactam-susceptible strains. Transformation of IS*Aba1*-*bla*_ADC-30_ into susceptible strains increased the sulbactam MIC from 2 to 32 μg/ml, which required *bla*_ADC-30_ overexpression using an upstream promoter in IS*Aba1*. Flow cytometry showed that ADC-30 production increased in response to sulbactam, ticarcillin, and ceftazidime treatment. This effect was regulated at the RNA level but not by an increase in the *bla*_ADC-30_ gene copy number as indicated by quantitative PCR. Purified ADC-30 decreased the inhibitory zone created by sulbactam or ceftazidime, similarly to TEM-1. In conclusion, ADC-30 overexpression conferred resistance to sulbactam in diverse clinical *A. baumannii* isolates.

## Introduction

*Acinetobacter baumannii* causes various nosocomial infections, and the prevalence of multidrug-resistant (MDR) *A. baumannii* has been increasing in different countries. This bacterium has intrinsic resistance to multiple drugs and can gain resistance mechanisms from other species (Peleg et al., 2008). The SENTRY program documented non-susceptibility to carbapenems, the last resort of drugs for the treatment of MDR *A. baumannii*, increased from 34.5% in 2006 to 59.8% in 2009 worldwide (Gales et al., 2011). In Taiwan, the rate of multidrug resistance in *Acinetobacter spp*. also increased from 1.3% in 2002 to 41.0% in 2010 (Kuo et al., 2012). In severely ill patients, infections with MDR isolates have been associated with high mortality due to the absence of appropriate or effective treatment options (Peleg et al., 2008). Combination therapies or new drugs such as antimicrobial peptides or silver nanoparticles have been proposed as novel modalities to treat MDR *A. baumannii* (Peleg et al., 2008; Tiwari et al., 2014)

Sulbactam is a β-lactamase inhibitor that is typically combined with penicillins because sulbactam lacks antimicrobial activity against most bacterial species (Adnan et al., 2013). However, sulbactam has demonstrated bacteriostatic or bactericidal effects against *A. baumannii* (Corbella et al., 1998). Combination treatment with sulbactam and carbapenems has shown promising *in vivo* and *in vitro* synergistic effects against MDR *A. baumannii* (Wolff et al., 1999; Ko et al., 2004; Song et al., 2007), and clinical success has been reported (Karageorgopoulos and Falagas, 2008). The addition of sulbactam to other antibiotics has been proposed in the treatment of MDR *A. baumannii*; however, the resistance rate to ampicillin/sulbactam in *Acinetobacter spp*. has increased to approximately 60% in certain area (Kuo et al., 2012).

Until recently, the mechanism underlying sulbactam resistance in *A. baumannii* was less commonly studied. In 2013, Krizova and colleagues demonstrated that the β-lactamase TEM-1 contributes to sulbactam resistance (Krizova et al., 2013), which led us to examine whether other selected β-lactamases found in *A. baumannii*, including the *Acinetobacter*-derived cephalosporinase (ADC), OXA-23, OXA-24/72, and OXA-58 families, also contribute to sulbactam resistance. Using clinical isolates collected from a Taiwanese surveillance program, we aimed to identify the β-lactamases associated with sulbactam resistance and to test the role of these β-lactamases in sulbactam resistance in *A. baumannii*.

## Materials and methods

### Association of selected β-lactamases with sulbactam resistance in *A. baumannii*

*A. baumannii* clinical isolates were randomly selected from the Taiwan Surveillance of Antimicrobial Resistance (TSAR) program, which contains 1640 *Acinetobacter* isolates collected from 2002 to 2010 (Kuo et al., 2012). *A. baumannii* was identified at the species level using multiplex PCR targeting the specific 16-23S rDNA intergenic spacer region (Chen et al., 2007). Pulsed-field gel electrophoresis was performed to determine clonality as previously described (Kuo et al., 2013). Isolates with similarity of >80% was designated as a single clone. The sulbactam-resistant and sulbactam-susceptible isolates were randomly selected and subjected to PCR testing for the presence of genes encoding ADC, OXA-23, OXA-24/72, and OXA-58 β-lactamases (Table S1). The PCR program (Krizova et al., 2013; Kuo et al., 2013) for genes encoding OXA was as followed: 94°C for 1 min, and 30 cycles of 25 s at 94°C, 40 s at 52°C and 50 s at 72°C; for PCR of *bla*_ADC_, 30 cycles of 60 s at 94°C, 60 s at 58°C, and 120 s at 72°C. GoTaq Flexi DNA polymerase (Promega, Madison, WI) was used for PCR assays performed in the GeneAmp PCR System 2700 (Applied Biosystems, Foster City, CA). Amplified DNA product was resolved by electrophoresis in agarose 2% w/v gels, stained with ethidium bromide, and purified according to the manufacturer's instruction (Geneaid Biotech Ltd, Taipei, Taiwan).

### Transformation of plasmids carrying different β-lactamase genes

Representative β-lactamase genes and their associated promoters, including *bla*_TEM−1_, *bla*_ADC_ and *bla*_OXA−23_ with their upstream insertion sequence IS*Aba1* (IS*Aba1*-*bla*_ADC_ and IS*Aba1-bla*_OXA−23_, respectively), *bla*_OXA−24/72_, and *bla*_OXA−58_ with its upstream IS*Aba3* that was truncated with IS*1008* (IS*1008*–ΔIS*Aba3-bla*_OXA−58_) (Chen et al., 2010), were PCR-amplified using the forward and reverse primers shown in Table S1. The PCR products were amplified with a proofreading DNA polymerase (Phusion High-Fidelity DNA Polymerase, Finnzymes, Espoo, Finland), cloned into the pCRII-TOPO vector (Invitrogen, Carlsbad, CA, USA) and subjected to sequencing (Mission Biotech, Taipei, Taiwan). The digested fragments were cloned into the *Xba*I and *Xho*I sites of the *Escherichia coli*-*A. baumannii* shuttle vector pYMAb2 (Kuo et al., 2013), which contains a kanamycin-resistant determinant. The fragment was cloned in-frame with a polyhistidine (His) tag, causing the resulting protein to be His-tagged. The recombinant plasmid and a control plasmid (pYMAb2 without β-lactamase genes) were transformed into the kanamycin-susceptible *A. baumannii* strain ATCC15151. ATCC15151 already contained *bla*_OXA−51_; therefore, *bla*_OXA−51_was not included in the experiment. Electroporation was performed with a gene pulser electroporator (Bio-Rad, Hercules, CA, USA) and 2-mm electrode gap cuvettes (Kuo et al., 2013). Transformants were selected based on kanamycin resistance, and sequencing was performed to confirm the presence of each β-lactamase gene.

### Antimicrobial susceptibility

Minimal inhibitory concentrations (MICs) of sulbactam, ceftazidime, ampicillin, imipenem, meropenem, and ticarcillin were determined by the agar dilution method according to the guidelines provided by the Clinical and Laboratory Standards Institute (CLSI) (Clinical and Laboratory Standards Institute, 2012). Sulbactam susceptibility parameters were adopted from the previous CLSI guidelines in which sulbactam MICs of less than 4 or more than 16 μg/ml were defined as susceptible or resistant, respectively.

### Immunofluorescent staining and enumeration by flow cytometry for protein expression

Immunofluorescent staining was performed as previously described with several modifications (Moe et al., 1999). Specifically, bacterial cultures were diluted in phosphate buffered saline (PBS) to ~10^8^ CFU/mL, and 0.5-ml samples were transferred into 1.5-ml Eppendorf microtubes. The samples were centrifuged at 5000 × *g* for 5 min, washed with 0.1% NaN_3_ PBS, and centrifuged for 5 min. The resulting bacterial pellet was resuspended in fixative (4% paraformaldehyde in PBS) for 20 min. The fixed samples were washed twice with quenching solution (100 mM NaCl, 50 mM Tris-HCl, pH 8.0) and resuspended in permeable buffer (1% Triton X-100, 0.1% NaN_3_ in PBS) for 5 min. After centrifugation, the samples were resuspended in 500 μL blocking buffer (1% BSA and 0.1% NaN_3_ in PBS).

To identify His-tagged ADC-30 expressed by ATCC15151 (pYMAb2::IS*Aba1*-*bla*_ADC−30_), each 100-μL sample was incubated with 2 μL of mouse anti-His-6-tag antibody (Sigma-Aldrich, St. Louis, MO, USA) at 4°C for 2 h. After washing by blocking buffer, the samples were stained for 1 h at 4°C with 5 μL of phycoerythrin (PE)-conjugated anti-mouse IgG antibody (Sigma-Aldrich). The stained samples were spun at 5000 × *g* for 5 min, and the cell pellet was resuspended in 500 μL of 1% paraformaldehyde buffer and stored at 4°C overnight. Cytometry samples were resuspended in PBS and analyzed in a flow cytometer system with wavelength of 575 nm (FACScanto II, BD Biosciences, San Jose, CA, USA).

### Quantitative PCR (qPCR) to determine the bla_ADC−30_ gene copy number after challenging with different antimicrobials

ATCC15151 (pYMAb2::IS*Aba1*-*bla*_ADC−30_) strains at mid-log phase were incubated in Luria-Bertani (LB) broth with different antimicrobial agents (25% of MIC) for 6 h. The *bla*_ADC−30_ copy number in these bacteria was estimated by qPCR using primers targeting *bla*_ADC−30_, and the housekeeping gene, *rec*A, was used as an internal control. Each qPCR reaction contained a total volume of 10 μL with 2 ng of genomic DNA as template, 100 nmol/L of each primer, and 1 × SYBR Green® PCR Master Mix (Applied Biosystems, Carlsbad, CA, USA) with ROX™ (Kapa Biosystems, Woburn, MA, USA). The relative *bla*_ADC−30_ copy number in the bacteria treated with different antimicrobial agents was normalized to the number found in the bacteria treated with LB broth without antimicrobial agents. The qPCR conditions included 2 min at 50°C (UNG activation), 10 min at 95°C, followed by 45 cycles of 15 s at 95°C and 1 min at 60°C. At the end, a dissociation stage was added: 15 s at 95°C, 15 s at 60°C, and 15 s at 95°C. All experiments were conducted using the ABI 7500 Fast Real-time PCR system (Applied Biosystems, Inc., Carlsbad, CA, USA) and were performed in triplicate.

### Quantitative reverse transcription PCR (qRT-PCR) to assess mRNA expression after challenging with different antimicrobials

After incubation with different antimicrobial agents, the ADC-30 mRNA levels in ATCC15151 (pYMAb2::IS*Aba1*-*bla*_ADC−30_) were compared using qRT-PCR (Chen et al., 2010). Briefly, around 2 μg of RNA was extracted with RNAprotect Bacteria Reagent and an RNeasy mini-kit (Qiagen, Valencia, CA, USA). Residual genomic DNA was removed using RNase-free DNase. The RNA was reverse transcribed into single-stranded cDNA with random hexamers and Moloney Murine Leukemia Virus reverse transcriptase (Epicenter, Madison, WI, USA). The cDNAs were subsequently quantified by real-time PCR amplification with conditions mentioned above. Expression level results were standardized to the transcription levels of *rpo*B gene for each strain, but relatively to the culture in LB (2 delta–delta Ct method). Negative controls without reverse transcription were performed to detect DNA contamination in the purified RNA.

### ADC-30 purification

His-tagged ADC-30 (in which the stop codon was deleted, and the proteins were fused with 6 His amino acids) expressed by ATCC15151 (pYMAb2::IS*Aba1*-*bla*_ADC−30_) was purified with Ni-NTA Superflow column (Qiagen). Briefly, the bacteria equal to ~10^7^ CFU/ml were centrifuged, resuspended in lysis buffer and sonicated. The lysate was diluted in binding buffer (25 mM Tris, 150 mM NaCl, 10 mM imidazole, pH 7.5) and loaded onto the column. The column was washed with five column volumes of wash buffer, and the protein was eluted with five column volumes of elution buffer (25 mM Tris, 150 mM NaCl, 300 mM imidazole). The protein solution was then dialyzed and concentrated by ultrafiltration on a 10 KDa-cutoff Amicon membrane (Millipore). The purity was assessed by sodium dodecyl sulfate-polyacrylamide gel electrophoresis (SDS-PAGE) and Western blotting as greater than 95%.

### SDS-PAGE and western blotting to detect purified β-lactamases

For SDS-PAGE and Western blotting (Blair et al., 2009), the purified protein was first separated by SDS-PAGE on 12% acrylamide gel using Mini-PROTEAN system (Bio-Rad) and transferred to a nitrocellulose membrane (PerkinElmer, Boston, MA, USA) in transfer buffer (25 mM Tris-HCl, 190 mM glycine, 20% methanol, 0.1% SDS, pH 8.3) at 350 mA for 1 h. The membranes were blocked with 10% non-fat dried milk in Tris-bufferred saline with 0.5% Tween-20. After hybridization with anti-His antibodies (Sigma-Aldrich) and peroxidase-conjugated goat anti-mouse secondary antibodies (Millipore, Temecula, CA, USA), the band was visualized with an ECL Western blot kit (PerkinElmer, Boston, MA, USA).

### Bioassays to confirm the activity of purified ADC-30 against ceftazidime and sulbactam

The first bioassay involved the mixture of 10 μL of purified ADC-30 or extracts of sulbactam- resistant or -susceptible strains with 10 μL of ceftazidime for 30 min at 37°C. Each 20-μL mixture was loaded onto a blank disk (Becton Dickinson, Sparks, MD, USA) that was placed in an agar plate containing a lawn of ceftazidime-susceptible *A. baumannii* ATCC 15151. Inhibitory zones were measured after incubating the plates overnight at 37°C.

The second bioassay conducted was similar to the modified Hodge test for carbapenemase detection. A 0.5 McFarland standard suspension of the sulbactam-susceptible *A. baumannii* ATCC 15151 strain was diluted to 1:10 and inoculated on an Mueller-Hinton agar plate for routine disk diffusion test. The 30-μg sulbactam disk was placed in the center of the plate, and the purified ADC-30, TEM-1, or PBS samples were drawn in a straight line out from the edge of the disk. The phenotype was evaluated after an overnight incubation at 37°C.

## Results and discussion

### Detection of selected β-lactamases in clinical *a. baumannii* isolates using a nationwide surveillance system

**Figure 1:**
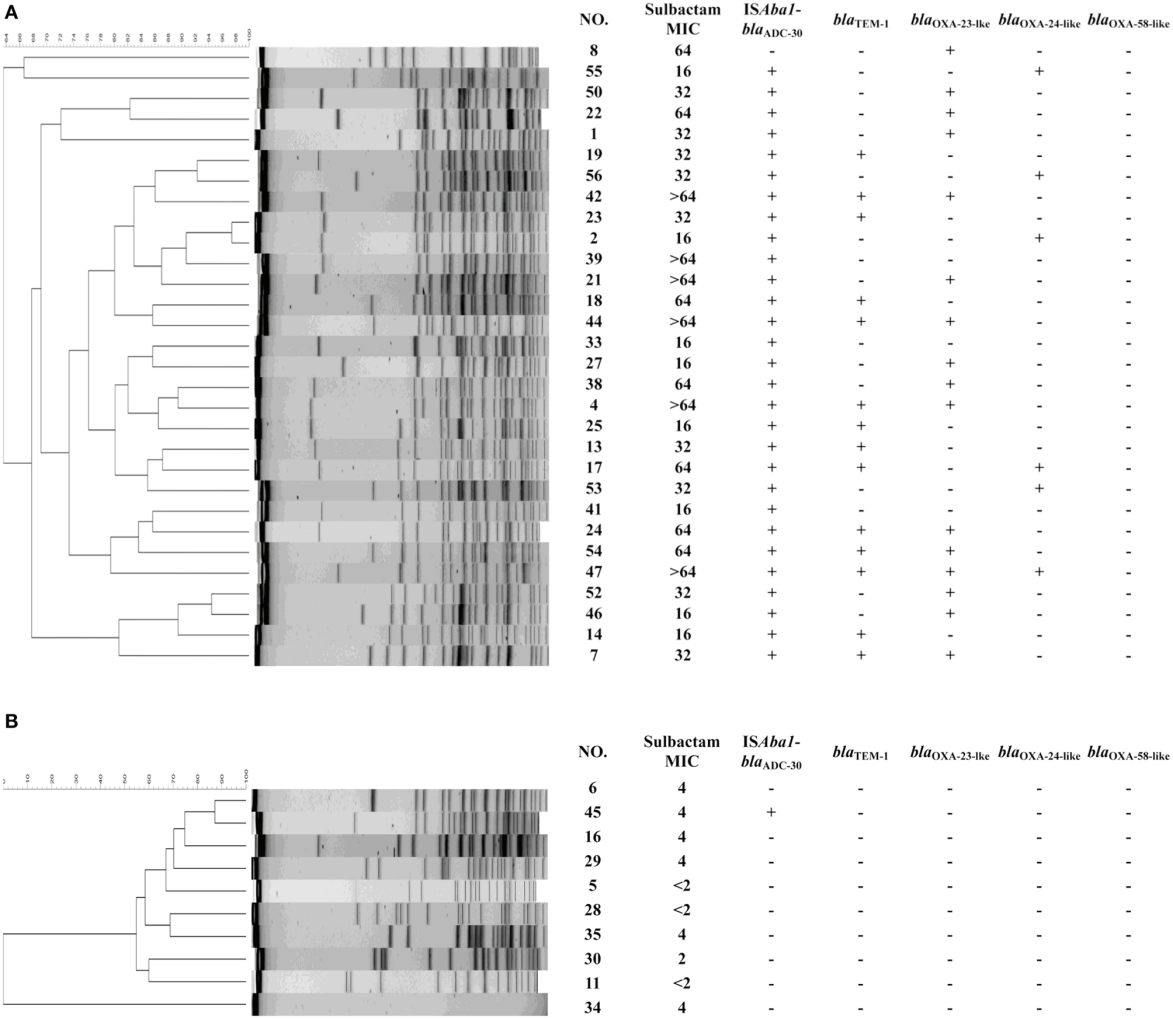
**Molecular characteristics of randomly selected *Acinetobacter baumannii* from the Taiwan Surveillance of Antimicrobial Resistance (TSAR) program, 2002–2010**. The results of pulsed-field gel electrophoresis are shown, followed by the minimal inhibitory concentrations (MICs), and the presence of IS*Aba1*-*bla*_ADC-30_, *bla*_TEM-1_, IS*Aba1-bla*_OXA-23-lke_, *bla*_OXA-24-like_, and *bla*_OXA-58-like_ in the **(A)** sulbactam-resistant strains and **(B)** sulbactam-susceptible strains.

Of the 30 sulbactam-resistant isolates tested, 14 (47%) were positive for *bla*_TEM−1_, and 29 (97%) possessed *bla*_ADC_ with IS*Aba1* upstream (IS*Aba1*-*bla*_ADC_). The PFGE shown in Figure 1 depicts the strain diversity. Based on clonality, 19 isolates belonging to different clones and carrying IS*Aba1*-*bla*_ADC_ were sent for sequencing. The *bla*_ADC_ sequences of all 19 strains were consistent with the *bla*_ADC−30_ sequence. IS*Aba1-bla*_OXA−23_, *bla*_OXA−24/72_ and IS*1008*–ΔIS*Aba3-bla*_OXA−58_ were present in16, 6, and 0 isolates, respectively. In 10 sulbactam-susceptible isolates, one possessed IS*Aba1*-*bla*_ADC−30_. None of the isolates possessed *bla*_TEM−1_. Therefore, in addition to TEM-1(Krizova et al., 2013), IS*Aba1*-*bla*_ADC−30_ may play a role in providing *A. baumannii* with sulbactam resistance.

The presence of one resistant strain without IS*Aba1*-*bla*_ADC_ nor *bla*_TEM−1_ indicates that another mechanism may be involved in sulbactam resistance. Various combinations of resistance mechanisms, including β-lactamase overexpression, the up-regulation of the efflux pump and the inactivation or down-regulation of porin, are often required for the development of β-lactam resistance in *Pseudomonas aeruginosa* and *A. baumannii* (Quale et al., 2006; Peleg et al., 2008; Tiwari et al., 2012; Tiwari and Moganty, 2014), which may explain the presence of IS*Aba1*-*bla*_ADC−30_ in one susceptible strain. However, the high rate of IS*Aba1*-*bla*_ADC−30_ in resistant strains and its low rate in susceptible strains (97% vs. 10%, Chi-square test *p* < 0.001) indicate the importance of IS*Aba1*-*bla*_ADC−30_ for the development of sulbactam resistance.

### Contribution of ADC-30 and IS*Aba1* to sulbactam resistance

To further confirm the association observed in the epidemiological survey, IS*Aba1*-*bla*_ADC−30_, *bla*_TEM−1_,IS*Aba1-bla*_OXA−23_, *bla*_OXA−24/72_ (and its promoter), and IS*1008*–ΔIS*Aba3-bla*_OXA−58_ were cloned and transformed into a sulbactam-susceptible reference strain, respectively. Shuttle vectors were also transformed, and changes in the MICs were measured (Table 1).

**Table 1:** **Minimal inhibitory concentrations (μg/ml) of *Acinetobacter baumannii* reference strain and different transformants**.

**Strains**	**Sulbactam**	**Ceftazidime**	**Ampicillin**	**Imipenem**	**Meropenem**	**Ticarcillin**
ATCC15151	2	8	64	0.25	0.5	16
ATCC15151 (pYMAb2)	2	8	64	0.25	0.5	16
ATCC15151 (pYMAb2::IS*Aba1*-*bla*_ADC-30_)	32	512	>1024	2	4	128
ATCC15151 (pYMAb2::*bla*_ADC-30_ Δ P1 [deletion of -35 promoter])	4	32	256	0.25	0.25	32
ATCC15151 (pYMAb2::*bla*_ADC-30_ Δ P2 [deletion of -10 and -35 promoter])	4	32	64	0.5	0.25	16
ATCC15151 (pYMAb2::IS*Aba1*-*bla*_OXA-23_)	16	8	>1024	16	16	>1024
ATCC15151 (pYMAb2::*bla*_*OXA*-24/72_)	4	8	1024	16	64	512
ATCC15151 (pYMAb2::IS*1008*–ΔIS*Aba3-bla*_*OXA*-58_)	8	4	>1024	16	8	>1024
ATCC15151 (pYMAb2:: *bla*_TEM-1_)	16	8	>1024	0.25	0.25	>1024
ATCC15151 (pYMAb2:: *bla*_TEM-1_ ΔP [deletion of promoter])	4	8	64	0.25	0.25	32

ATCC15151 (pYMAb2::IS*Aba1*-*bla*_ADC−30_) exhibited the highest increases of sulbactam MIC (16-fold). Other β-lactamases, including OXA-23, OXA-72, and OXA-58, contributed to the increase in the sulbactam MICs, although at lower levels. IS*Aba1*-*bla*_ADC−30_ was used in the study due to the high level of sulbactam resistance and high prevalence of IS*Aba1*-*bla*_ADC−30_, which were comparable to the sulbactam resistance levels and prevalence of *bla*_TEM−1_.

The regulatory mechanism for ADC expression in *Acinetobacter* spp. may be different from the mechanism in many *Enterobacteriaceae* because *Acinetobacter* spp. lack the *Amp*R gene (Jacoby, 2009). The presence of IS*Aba1* upstream of *bla*_AmpC_ is essential for ceftazidime resistance due to the AmpC overexpression (Heritier et al., 2006). To confirm the role of the promoter located within IS*Aba1* in mediating sulbactam resistance, plasmids harboring *bla*_ADC−30_ without upstream -35 (within IS*Aba1*) or -35/-10 promoters were transformed into ATCC15151. The MICs increased by only 2-fold compared with the control (Table 1). Therefore, as previous studies have indicated (Heritier et al., 2006), these promoters in the IS*Aba1* are required for ADC-mediated sulbactam resistance. Similarly, deletion of the promoter upstream of *bla*_TEM−1_ decreased the sulbactam MIC.

### ADC-30 production in response to treatment with sulbactam and other substrates

**Figure 2:**
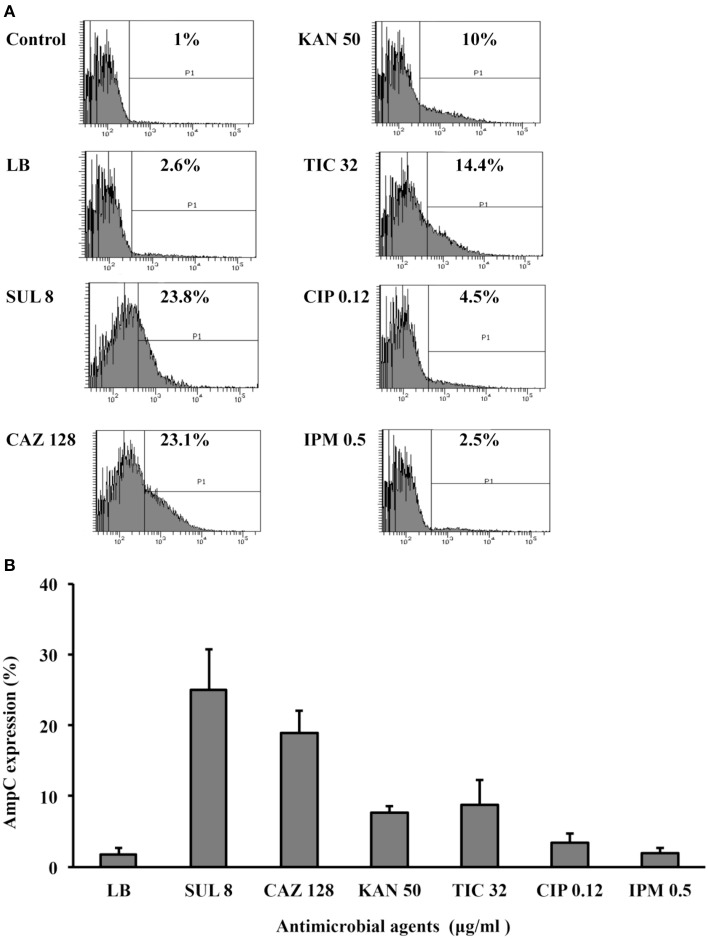
**ADC-30 production in response to treatment with sulbactam and other substrates. (A)** Flow cytometry showed that the ADC-30 protein expression level increased in response to its substrates (ceftazidime, sulbactam, and ticarcillin) compared with the level observed in bacteria cultured in Luria-Bertani (LB) broth without antimicrobial agents. The tests were performed in triplicate; however, only one of the representative experiments is shown. **(B)** Quantified values of experiments performed in triplicate are shown in the bar graph. LB broth supplemented with kanamycin was used as a positive control because pYMAb2 also carries a kanamycin resistance determinant. CAZ, ceftazidime; CIP, ciprofloxacin; IPM, imipenem; KAN, kanamycin; SUL, sulbactam.

The production of β-lactamase usually increases in response to its substrates; therefore, we asked whether the addition of sulbactam increases ADC-30 production. Flow cytometry (Figure 2) showed that ADC-30 protein expression increased significantly after the addition of sulbactam or ceftazidime compared with the control. ADC-30 production was also induced by ticarcillin but not by ciprofloxacin or imipenem. The results indicate that the co-selection of sulbactam resistance by other antimicrobial agents occurs.

**Figure 3:**
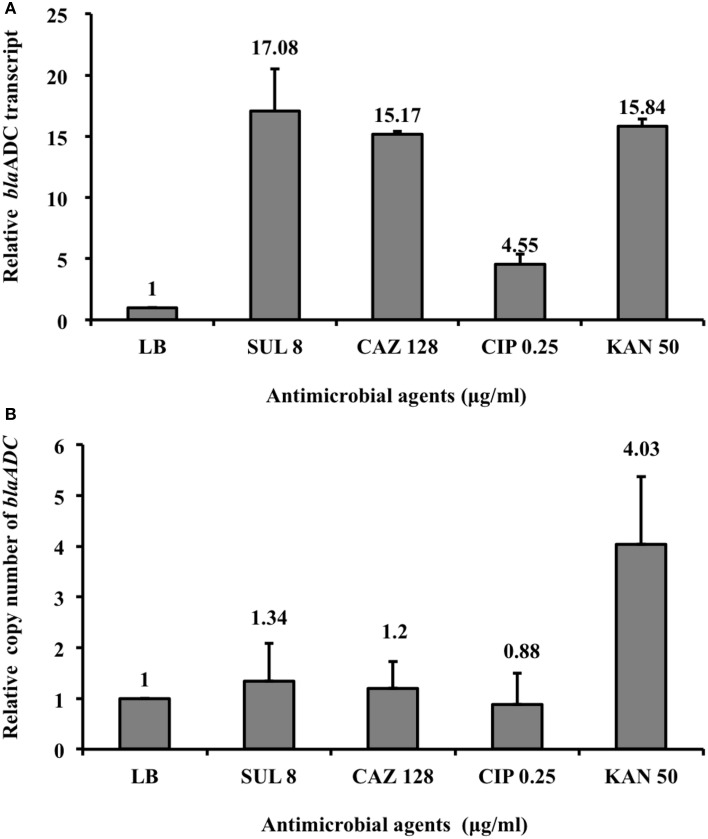
***Bla*_ADC-30_ mRNA expression level and gene copy number in response to treatment with sulbactam and other antimicrobial agents. (A)** The *bla*_ADC-30_ mRNA expression level increased after treatment with sulbactam and ceftazidime. ATCC15151 (pYMAb2::IS*Aba1*-*bla*_ADC-30_) treated with ciprofloxacin or without antimicrobial agents were used as negative controls. **(B)** The *bla*_ADC-30_ gene copy number did not differ, regardless of the antimicrobial agents added. The mRNA expression level increased in response to kanamycin because of the increased number of plasmids (pYMAb2) carrying the kanamycin-resistant gene. The test was performed in triplicate. CAZ, ceftazidime; CIP, ciprofloxacin; KAN, kanamycin; LB, Luria-Bertani broth; SUL, sulbactam.

The qRT-PCR results (Figure 3) showed that ADC-30 mRNA expression increased in response to its substrates (ceftazidime and sulbactam) but not in response to other antimicrobial agents (ciprofloxacin). Carbapenemase expression has been related to the high *bla*_OXA−58_ plasmid copy number (Bertini et al., 2007; Chen et al., 2008). Therefore, we asked whether the increased ADC-30 protein and mRNA expression levels in the presence of sulbactam or ceftazidime are attributed to an increase in the *bla*_ADC−30_ copy number. The qPCR results (Figure 3) showed no differences in the *bla*_ADC−30_ copy numbers of cells treated with sulbactam or ceftazidime compared with the negative controls. In contrast, the gene copy number increased in response to kanamycin. The results indicate that the increase in ADC-30 was regulated at the RNA level.

How the addition of sulbactam increased ADC-30 protein and mRNA expression level in *A. baumannii* is unknown. The induction of AmpC by β-lactams has been commonly described in *P. aeruginosa* and many *Enterobacteriaceae* (Jacoby, 2009). After the treatment of β-lactams, altered peptidoglycan synthesis leads to increased expression of AmpC through AmpG–AmpR–AmpC pathway (Zeng and Lin, 2013). Clavulanate, another β-lactamase inhibitor with structure similar to β-lactams, also induces the expression of AmpC in many *Enterobacteriaceae* (Drawz and Bonomo, 2010). Although sulbactam may interfere the wall synthesis by interacting with penicillin-binding protein 1 and 3 (Penwell et al., 2015), lack of *Amp*R in *A. baumannii* indicated other mechanisms, rather than AmpG–AmpR–AmpC pathway, are responsible. The two-component system has been proposed to be involved in the induction of AmpC and other chromosomally encoded β-lactamases (Jacoby, 2009; Zeng and Lin, 2013). However, the mechanism regarding the induction of ADC in response to sulbactam in *A. baumannii* requires further investigation.

### Contribution of purified ADC-30 to sulbactam resistance

**Figure 4:**
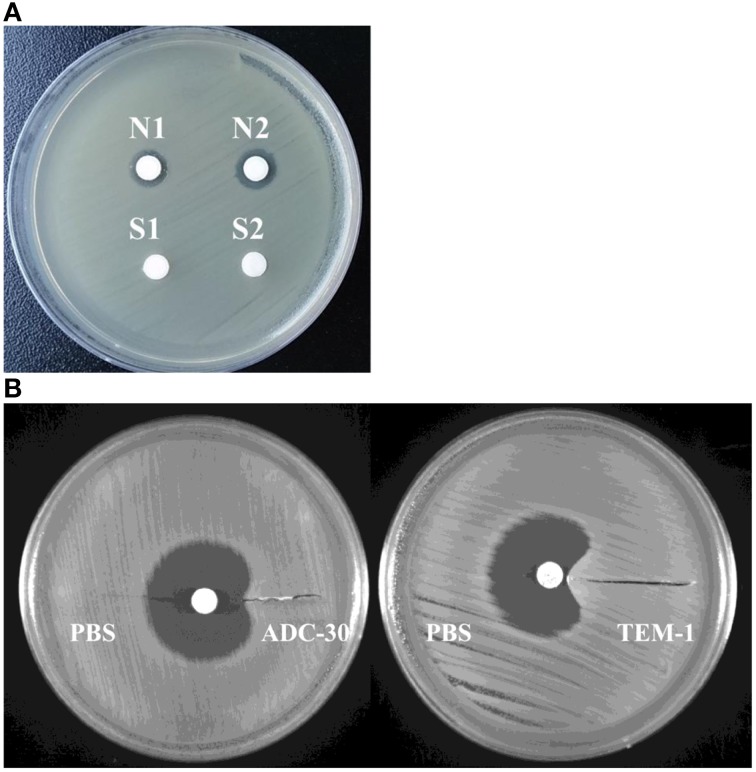
**Activity of ADC-30 against ceftazidime and sulbactam. (A)** ADC-30 remained active against ceftazidime in *Acinetobacter baumannii* after purification. Disks containing ceftazidime and different samples were placed in an agar plate containing a lawn of ceftazidime-susceptible *A. baumannii* ATCC 15151. Crude extract of the ATCC15151 (pYMAb2::IS*Aba1*-*bla*_ADC-30_) strain and purified ADC-30 (denoted as S1 and S2 in the lower filed) promoted the growth of the *A. baumannii* ATCC 15151 strain against disks loaded with ceftazidime. Controls included N1 (phosphate buffered saline) and N2 [crude extract of ATCC15151 (pYMAb2)], with which the ceftazidime created an inhibitory zone. **(B)** The bioassay resembling modified the Hodge test showed that purified ADC-30 and TEM-1 both promoted *A. baumannii* ATCC 15151 growth against sulbactam. PBS, phosphate buffered salinex.

ADC-30 was purified (Figure S1), and the addition of purified ADC-30 to the disk decreased the inhibitory zone caused by ceftazidime (Figure 4A), confirming the activity of the purified protein. The bioassay resembling the modified Hodge test showed that purified ADC-30 promoted the inward growth of sulbactam-susceptible *A. baumannii* similarly to purified TEM-1 (Figure 4B). Therefore, ADC-30 may directly interact with sulbactam and confer sulbactam resistance.

In conclusion, *bla*_ADC−30_ overexpression contributes to sulbactam resistance in *A. baumannii*, which is prevalent in clinical isolates of different clones in Taiwan over a long period of time. The resistance mechanisms are induced at the mRNA and protein levels by other antimicrobial agents in addition to sulbactam, supporting the cautious use of these antibiotics to avoid the selection of sulbactam-resistant *A. baumannii* isolates.

## Ethical approval

This study was approved by the Institutional Review Board of National Health Research Institutes.

### Conflict of interest statement

Te-Li Chen is a medical advisor of TTY Biopharm. The authors declare that the research was conducted in the absence of any commercial or financial relationships that could be construed as a potential conflict of interest.
