# Supplementary material for: Contribution of Acinetobacter-derived cephalosporinase-30 to sulbactam resistance in Acinetobacter baumannii
Source: Front Microbiol. 2015 Mar 25;6:231. doi: 10.3389/fmicb.2015.00231 (PMC4517069; doi:10.3389/fmicb.2015.00231)

Figure S1. SDS-PAGE and Western blot of purified ADC-30 at different purification steps. C, crude extract of ATCC15151 (pYMAb2::IS*Aba1*-*bla*$_{ADC-30}$); F, fraction during flow-through; M, marker; N, negative control, crude extract of ATCC15151 (pYMAb2); W, fraction during washing step; No 1-11, 15 and 20, eluted fractions during the corresponding elution step.

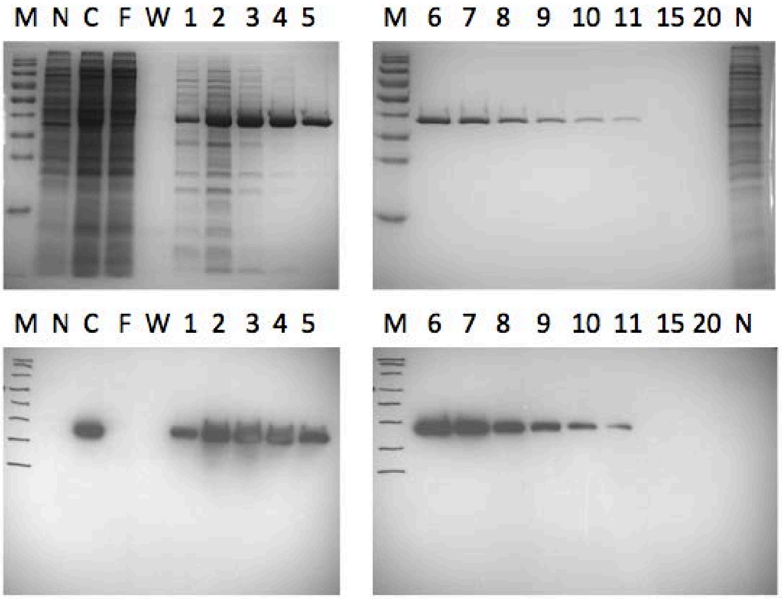

Supplement: Supplementary file 1 [file Image1.PDF]
