# Supplementary material for: Contribution of Acinetobacter-derived cephalosporinase-30 to sulbactam resistance in Acinetobacter baumannii
Source: Front Microbiol. 2015 Mar 25;6:231. doi: 10.3389/fmicb.2015.00231 (PMC4517069; doi:10.3389/fmicb.2015.00231)
Supplement: Supplementary file 2 [file Table1.PDF]

Table S1. Primers used in this study

**Primers**	**Sequences, 5′–3′**
IS1008(*Xba*I)F	tctagaTCTATTTGCAACAGTGCCATTTTTCTT
OXA-58 (*Xho*I)R	ctcgagTTATAAATAATGAAAAACACC
ISAba1(*Xba*I)F	tctagaCACGAATGCAGAAGTTG
OXA-23-like(*Xho*I)R	ctcgagAATAATATTCAGCTGTTTTAATG
OXA-24(*Xba*I)F	tctagaCTCTAAGCCCCAAAATTTCC
OXA-24(*Xho*I)R	ctcgagAATGATTCCAAGATTTTCTAGCG
AmpC(*Xho*I)R	ctcgagTTTCTTTATTGCATTCAGCAC
TEM-1 (*Xba*I)F	tctagaATAAAATTCTTGAAG
TEM-1 (*Xho*I)R	ctcgagTTACCAATGCTTAAT
TEM-A	TAAAATTCTTGAAGACG
TEM-B	TTACCAATGCTTAATCA
qPCR for ADC-30 (F)	ACGCCTGGTAAGTATTGGAAAG
qPCR for ADC-30 (R)	ACCGATTGAGTTTTTAGGTTTCC
qPCR for *rec*A (F)	TGAAGGCACATGTACCACCAG
qPCR for *rec*A (R)	ACCAAAAGGCCGTATTATCG
qPCR for *rpo*B (F)	GAGTCTAATGGCGGTGGTTC
qPCR for *rpo*B (R)	ATTGCTTCATCTGCTGGTTG
